# D1398G Variant of MET Is Associated with Impaired Signaling of Hepatocyte Growth Factor in Alveolar Epithelial Cells and Lung Fibroblasts

**DOI:** 10.1371/journal.pone.0162357

**Published:** 2016-09-01

**Authors:** Ilia Atanelishvili, Yuichiro Shirai, Tanjina Akter, Atsushi Noguchi, Kurt T. Ash, Suniti Misra, Sibnath Ghatak, Richard M. Silver, Galina S. Bogatkevich

**Affiliations:** 1 Division of Rheumatology and Immunology, Department of Medicine, Medical University of South Carolina, Charleston, South Carolina, United States of America; 2 Department of Allergy and Rheumatology, Nippon Medical School, Tokyo, Japan; 3 Department of Regenerative Medicine and Cell Biology, Medical University of South Carolina, Charleston, South Carolina, United States of America; Osaka University Graduate School of Medicine, JAPAN

## Abstract

Pulmonary fibrosis represents the terminal stage of a diverse group of lung diseases including scleroderma associated interstitial lung disease. The molecular mechanisms underlying the pathogenesis of lung fibrosis are not well understood and there is a great need for more effective treatment for this lethal disease. We recently discovered a small fragment of hepatocyte growth factor (HGF) receptor MET as a peptide designated “M10,” with strong antifibrotic properties. Furthermore, we showed that aspartic acid at position 1398 of MET is essential for M10 generation. The current study was undertaken to investigate the D1398G variant of MET in which aspartic acid at position 1398 was mutated to glycine resulting in loss of M10. We demonstrate that lung fibroblasts, A549, and primary alveolar epithelial cells (AEC) expressing D1398G MET exhibit reduced auto-phosphorylation on tyrosine residues and reduced activation of Ras and MAPK. HGF treatment of scleroderma lung fibroblasts as well as HGF treatment of TGFβ-treated normal lung fibroblasts transfected with wild type MET is associated with decreased collagen, connective tissue growth factor (CTGF, CCN2) and smooth muscle α-actin (SMA). However, HGF has no such effects in cells transfected with MET D1398G. Cisplatin- and FasL-induced apoptosis is significantly reduced in AEC transfected with MET wild type, but not in AEC transfected with MET D1398G. We conclude that the D1398G variant of MET is associated with compromised phosphorylation and impaired HGF signaling in lung fibroblasts and AEC, two cell types implicated in the pathogenesis of pulmonary fibrosis associated with scleroderma. Ongoing studies will explore the frequency of this variant and its relationship to pulmonary outcomes in scleroderma patients.

## Introduction

Systemic sclerosis (SSc; scleroderma) is a multi-system fibrotic disorder that affects skin and internal organs. Interstitial lung disease (ILD) or pulmonary fibrosis is a major organ complication and a leading cause of mortality and morbidity in SSc [1–3]. In particular, African American SSc patients exhibit higher prevalence of ILD and worse outcomes than those of other races [4–8]. Although recent studies have provided some molecular basis for such racial differences, the exact mechanisms of this important health disparity remain to be elucidated [9].

We previously reported that a cell-protective and antifibrotic factor, hepatocyte growth factor (HGF), is down-regulated in bronchoalveolar lavage fluid and plasma from African American SSc-ILD patients compared with Caucasian SSc-ILD patients [10]. Additionally, we demonstrated that antifibrotic effects mediated by the HGF receptor, also known as cellular mesenchymal-epithelial transition factor (c-MET, MET), are impaired in lung fibroblasts isolated from a subset of scleroderma patients with severe ILD suggesting a potential link between SSc-ILD and MET dysfunction [10].

MET is a transmembrane protein with structural features of tyrosine kinase receptor [11, 12]. MET contains the 50 kDa α-chain and the 140 kDa β-chain subunits, and the β-chain subunit comprises an extracellular part, a membrane spanning region, an intracellular C-terminal region that contains the tyrosine kinase domain, and two tyrosine multifunctional docking sites in the C-terminal tail [13, 14]. Whereas the mature form of MET is composed of 1408 amino acids, multiple MET transcripts of different sizes were identified. An isoform lacking 18 amino acids in the extracellular region called 1390 amino acid-isoform is believed to be the most abundant form in a variety of tissues and cell lines [13].

In response to HGF binding, MET undergoes autophosphorylation at tyrosine residues in the kinase domain (Y1234 and Y1235 in the 1390 amino acid-isoform) [15]. Subsequently, autophosphorylation activates phosphorylation of tyrosine residues in the multifunctional docking sites (Y1349 and Y1356), and these sites recruit multiple adaptor proteins, resulting in initiation of signal transduction [15].

MET is mainly expressed in epithelial and endothelial cells mediating potent mitogenic, motogenic, morphogenic, and anti-apoptotic effects of HGF in these cells [11–15]. MET is also expressed by myofibroblasts, where HGF exerts an anti-fibrotic effect and preserves organ function in bleomycin-induced lung fibrosis models [16–19]. MET, when overexpressed, has been reported to activate endogenous cysteine-dependent aspartate-directed proteases (caspases) following stress conditions in several cell lines [20–22]. Activated caspase-3 recognizes aspartic acid-containing motifs within MET and, in turn, cleaves MET [21, 22]. Such cleavage generates several stable fragments of the MET receptor that have been implicated in the regulation of cell apoptosis and MET expression [22]. One of the caspase-recognized motifs, DEV**D-**T, requires aspartic acid at position 1398 (1380 in alternatively spliced MET), shown above in bold. Mutation of 1398 aspartic acid to glycine prevents this site of MET from being recognized and cleaved by caspase-3, which suggests that the D1398G variant of MET is not able to generate the terminal 10-amino-acid-fragment, TRPASFWETS, designated in our laboratory as “M10” [23].

Recently, we demonstrated strong antifibrotic properties of M10 *in vitro* and *in vivo* [23]. The present study was designed to investigate effects of the D1398G mutation on HGF-induced functions of MET in lung fibroblasts (LF) and lung epithelial cells.

## Materials and Methods

### Lung Tissue and Cell Culture

The research presented in this manuscript is qualified as "Not Human Subjects Research" in accordance with the MUSC IRB. All de-identified specimens were received from the MUSC Multidisciplinary Clinical Research Center under the research proposal Pro00021985. Lung tissues were collected postmortem from three SSc patients who fulfilled the 2013 ACR/EULAR classification criteria for SSc [24] and had evidence of lung involvement. The diagnosis of SSc-ILD was confirmed by histological examination of postmortem lung tissue. Lung fibroblasts were isolated from scleroderma lung tissue and from age-, race-, and sex-matched controls as previously described [25, 26] and used between third and six passages in all experiments. Primary alveolar epithelial cells (AEC) type II were isolated from scleroderma lung tissue by dispase method [27]. Briefly, lung tissue was washed five times with PBS, saturated with dispase, and incubated at 37°C for 40 minutes. After incubation, lung tissue was transferred into 100-mm plate, dissected into very fine pieces, filtered by 100mm-40mm-20mm cell strainers, and resuspended in 5 ml of epithelial cell media (F12+10% FBS+1%AA). The cells were placed into 100 mm dish coated by CD45+CD32 antibodies and incubated for 90 minutes. The supernatant containing AEC type II was collected and placed in 4-chamber slides or 6 well plates for culturing. Human fetal lung fibroblasts MRC5 were purchased from Sigma (St. Louis, MO), human lung epithelial cells A549 were purchased from Lonza (Walkersville, MD) and from American Type Culture Collection (Manassas, VA), primary human pulmonary alveolar epithelial cells were obtained from ScienCell Research Laboratories (Carlsbad, CA).

### Generation of recombinant MET wild type and MET D1398G adenovirus plasmids

The wild type (WT) full-length MET cDNA in pLXSN was a generous gift from Dr. Morag Park, McGill University, Montreal, Canada. The Quick Change Lightning Multi Site-Directed Mutagenesis Kit (Agilent technologies, Santa Clara, CA) was used to generate MET D1398G mutant using forward primer GAAGATAACGCTGATGATGAGGTGGGCACACGAC CAG and reverse primer CTGGTCGTGTGCCCACCTCATCATCAGCGTTATCTTC. Xho-I and Hind-III were used to digest pLXSN+MET construct followed by subcloning of MET WT and MET D1398G into pAdTrack-CMV vector and digestion with Pac-I. The linearized plasmids were amplified in 293A cells according to the manual for the AdEasy system (Stratagene, La Jolla, CA) and purified by double CsCl gradient ultracentrifugation. The structure of MET WT and MET D1398G in pAdTrack-CMV was verified by nucleotide sequence analysis (Genewiz, South Plainfield, NJ). The titer, cytopathic effects and function of MET WT and MET D1398G recombinant adenoviruses were determined followed by adjusting virus particle per cell in both, lung fibroblasts and alveolar epithelial cells (S1 Fig). Western blots and immunofluorescent staining were routinely performed to monitor expression level changes of MET between infected and uninfected cells.

### RNA isolation and RT-PCR analysis

A549 cells were cultured in 10% FBS or in serum-free medium for 24 hours and subjected to total RNA extraction with the RNA Isolation Kit from QIAGEN (Valencia, CA) according to manufacturer’s recommendations. RNA purity and amount isolated was determined by spectrophotometric analysis. Reverse transcription was performed with the SuperScript II First-Strand Synthesis Kit from Invitrogen (Carlsbad, CA) and RT-PCR was performed with SYBR Green PCR Master Mix Kit from Bio-Rad (Hercules, CA). PCR primers were as follows: Pro-surfactant protein C (pro-SPC) forward CTTCTCCATCGGCTCCACTG, pro-SPC reverse GAGCCTCAAGACTGGGGATG; Glyceraldehyde-3-phosphate dehydrogenase (GAPDH) forward GGAAGGTGAAGGTCGGAGTC; GAPDH reverse TGGAATTTGCCATGGGTGGA. RT-PCR was performed on a Bio-Rad MyIQ single color Real-Time PCR detection system under the following conditions: 95°C for 3 min, followed by 35 cycles at 95°C for 30 sec and 60°C for 1 min. Relative differential gene expression of pro-SPC was calculated using the method described by Pfaffl [28] with GAPDH serving as a housekeeping gene. Real-time amplification plots of pro-SPC and GAPDH are presented in S2 Fig. Product size of the pro-SPC-specific transcript was confirmed by agarose gel (S3 Fig).

### Immunohistochemistry and immunofluorescent studies

Lung tissues were washed with PBS, fixed in 4% paraformaldehyde, and embedded in paraffin blocks. Seven μm paraffin sections were collected on slides, deparaffinized in histo-clear, and rehydrated through a degrading series of ethanol before staining. Antigen retrieval was performed by Antigen Unmasking solution (Vector Laboratories, Burlingame, CA) and permeabilized for 10 minutes in 0.1% Triton X-100. Nonspecific binding sites were blocked for 40 minutes in Background Buster (Innova Biosciences, Cambridge, UK). The slides were immunostained with anti-MET C12 from Santa Cruz Biotechnology (Santa Cruz, CA) and anti-MET 4F8.2 from EMD Millipore (Billerica, MA). Fluorescence signals were visualized with a Leica DMI4000B fluorescence microscope equipped with Hamamatsu Camera Controller ORCA-ER and quantified by Adobe Photoshop CS3 software using the Count Tool. Cells stained positively for MET were counted using at least six none-overlapping high-power fields at x400 magnification per sample. The results are presented as a percentage of positive cells over total nucleated cells. Lung tissues stained by hematoxylin and eosin were visualized by the Olympus BX40F4 light microscope with U-PMTVC Camera Adapter.

Cells were cultured to subconfluence on glass slides, fixed with 4% formaldehyde and blocked with PBS containing 5%BSA, 0.1%Triton, and 0.0004% Sodium Azide. The slides were immunostained with rabbit polyclonal pro-surfactant protein C antibody, anti-MET C12 antibody or with anti-MET 4F8.2 antibody followed by Alexa Fluor 647^®^ conjugated goat anti-rabbit secondary antibody. Next, slides were washed with PBS and incubated with Alexa Fluor 488^®^ phalloidin for 30 minutes (dilution in PBS 1:50). The labeled slide was mounted with ProLong Gold anti-fade reagent with DAPI and visualized under Zeiss Axio Imager M2 microscope system.

### Caspase 3 Assay

Caspase 3 Assay Kit (Abcam, Cambridge, MA) was used to detect apoptosis in cultured cells. The cells were plated on 100 mm plates, transfected with MET WT or D1398G, and treated with or without FasL and cisplatin for 24h. Cell lysates were prepared in accordance with the manufacturer’s instructions, transferred to a 96-well plate, and incubated with DEVD-p-NA substrate at 37°C for 2 hours followed by reading the absorbance at 405 nm on a plate reader.

### Ras Activity Assay

G-LISA Kit (Cytoskeleton Inc., Denver, CO) was used to measure endogenous Ras-GTP levels. Cells were plated on 100 mm plates, transfected with MET WT or variant D1398G, treated with or without HGF for 24h, and subjected to the assay in accordance with the manufacturer’s instructions. Bound Ras-GTP levels were determined by anti-Ras primary antibody followed by horseradish peroxidase-conjugated secondary antibody. A peroxidase substrate was applied, and the plates were read at 490 nm on a spectrophotometer.

### Preparation of cell extracts and immunoblotting

Cells were collected and analyzed by immunoblotting as previously described [10, 25]. Phosphorylation of MET was analyzed using anti-phospho-c-Met [pYpYpY^1230/1234/1235^], pY^1349^, and pY^1356^ antibodies (from Life Technologies (Miami, FL), Cell Signaling Technology (Danvers, MA), and Sigma-Aldrich (St. Louis, MO) respectively). Total MET was immunobloted using anti-MET (C12) from Santa Cruz Biotechnology (Santa Cruz, CA), anti-MET (25H2) was from Cell Signaling Technology (Danvers, MA), and pro-surfactant protein C antibody was from Seven Hills Bioreagents (Cincinnati, OH). Anti-type I collagen antibody (Southern Biotechnology, Birmingham, AL), anti-CTGF antibody (Santa Cruz Biotechnology, Santa Cruz, CA), and anti-smooth-α-actin antibody (Sigma-Aldrich, St. Louis, MO) were also used.

The phosphorylation of p42/p44 MAPK isoforms and total Erk1/2 levels were analyzed using anti-phospho-Erk1/2 and Erk1/2 antibodies in accordance with the manufacturer’s instructions (Cell Signaling Technology, Danvers, MA). Immunoblots were routinely stripped and re-blotted with anti-β-actin antibody (Sigma-Aldrich, St. Louis, MO) as a loading control.

### Statistical Analysis

Statistical analyses were performed with KaleidaGraph 4.0 (Synergy Software, Reading, PA). All data were analyzed using ANOVA with post-hoc testing. The results were considered significant if p<0.05.

## Results

### Expression of MET WT and MET D1398G mutant in different cell lines

**Fig 1:**
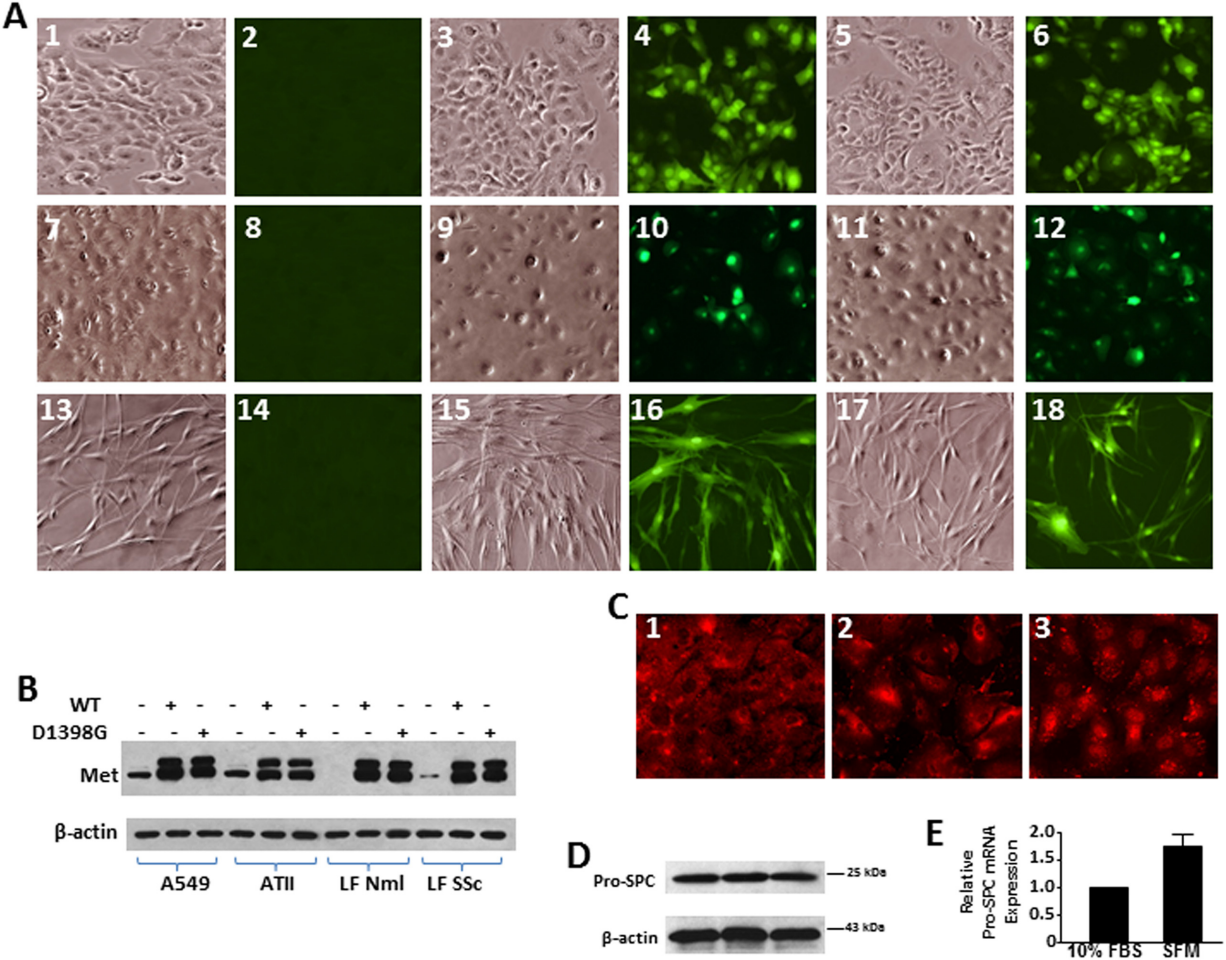
Transfection efficiency of MET WT and MET D1398G adenoviruses in different cell lines. **A.** A549 cells (images 1 through 6), ATII cells (images 7 through 12), and lung fibroblasts (images 13 through 18) were seeded on slides and transfected with empty adenovirus (without green fluorescent protein, GFP, images 1, 2, 7, 8, 13, 14), MET wild type (images 3, 4, 9, 10, 15, 16) and MET D1398G (images 5, 6, 11, 12, 17, 18) adenoviruses linked with GFP. In 48 hours after transfection, cells were fixed and subjected to a phase-contrast (images 1, 3, 5, 7, 9, 11, 13, 15, 17) and fluorescent (images 2, 4, 6, 8, 10, 12, 14, 16, 18) microscopy. **B.** Immortal alveolar epithelial cells A549, primary alveolar epithelial cells type II (ATII), normal (Nml) and scleroderma (SSc) lung fibroblasts (LF) were transfected with empty adenovirus or either with MET wild type and MET D1398G adenoviruses. In 48 hours after transfection, cells were collected with lysis buffer and analyzed by Western blot with anti-MET antibody as a transfection efficiency control and anti-β-actin antibody as a loading control. **C and D.** Pro-surfactant protein C expression in primary alveolar epithelial cells type II (ATII, image 1 and lane 1), primary human pulmonary alveolar epithelial cells (ScienCell Research Laboratories, image 2 and lane 2), and alveolar epithelial cells A549 (image 3 and lane 3). For immunofluorescent study **(C)**, cells were seeded into 4-chamber slides (40 000 cells per chamber) and cultured until 90% of confluence in F12 regular medium followed by overnight incubation in serum-free F12 medium. Next, cells were fixed by 4% formaldehyde and stained with anti-pro-surfactant protein C antibody (Seven Hills Bioreagents) followed by gout anti-rabbit secondary antibody. For Western blot **(D)**, cells were cultured in 6-well plates and subjected to immunoblotting as detailed in Materials and Methods. **E. Validation of pro-surfactant protein C (pro-SPC) expression in A549 cells by real-time RT-PCR.** The expression level of pro-SPC in A549 cells incubated in 10% FBS or serum-free medium (SFM) was normalized to GAPDH and presented as mean ± SD from two independent experiments.

Transfection of all studied cell lines with adenoviruses carrying MET WT and MET D1398G mutant was adjusted to multiplicity of infection 10 to 12 adenoviruses per one cell (S1 Fig). Expression of MET WT and MET D1398G mutant was routinely monitored by immunofluorescent staining and immunoblotting (Fig 1A and 1B). Lung fibroblasts used in this study were described in details in our previous publications [23, 27]. In the current study, in addition to the primary alveolar epithelial cells (AEC) type II, we used immortal A549 cells. The A549 cell line was established in 1972 from a human alveolar cell carcinoma. These cells are characterized by several features typical for alveolar epithelial cells type II, such as lamellar bodies in cytoplasm and pattern of phospholipids necessary for synthesis of pulmonary surfactant [29]. We previously reported that A549 cells incubated in serum free medium synthesize surfactant protein C [27]. Here we demonstrate that A549 cells contain pro-surfactant protein C similarly to primary AEC type II and commercially available primary AEC (Fig 1C, 1D and 1E).

### MET phosphorylation in LF and AEC transfected with MET WT and MET D1398G

**Fig 2:**
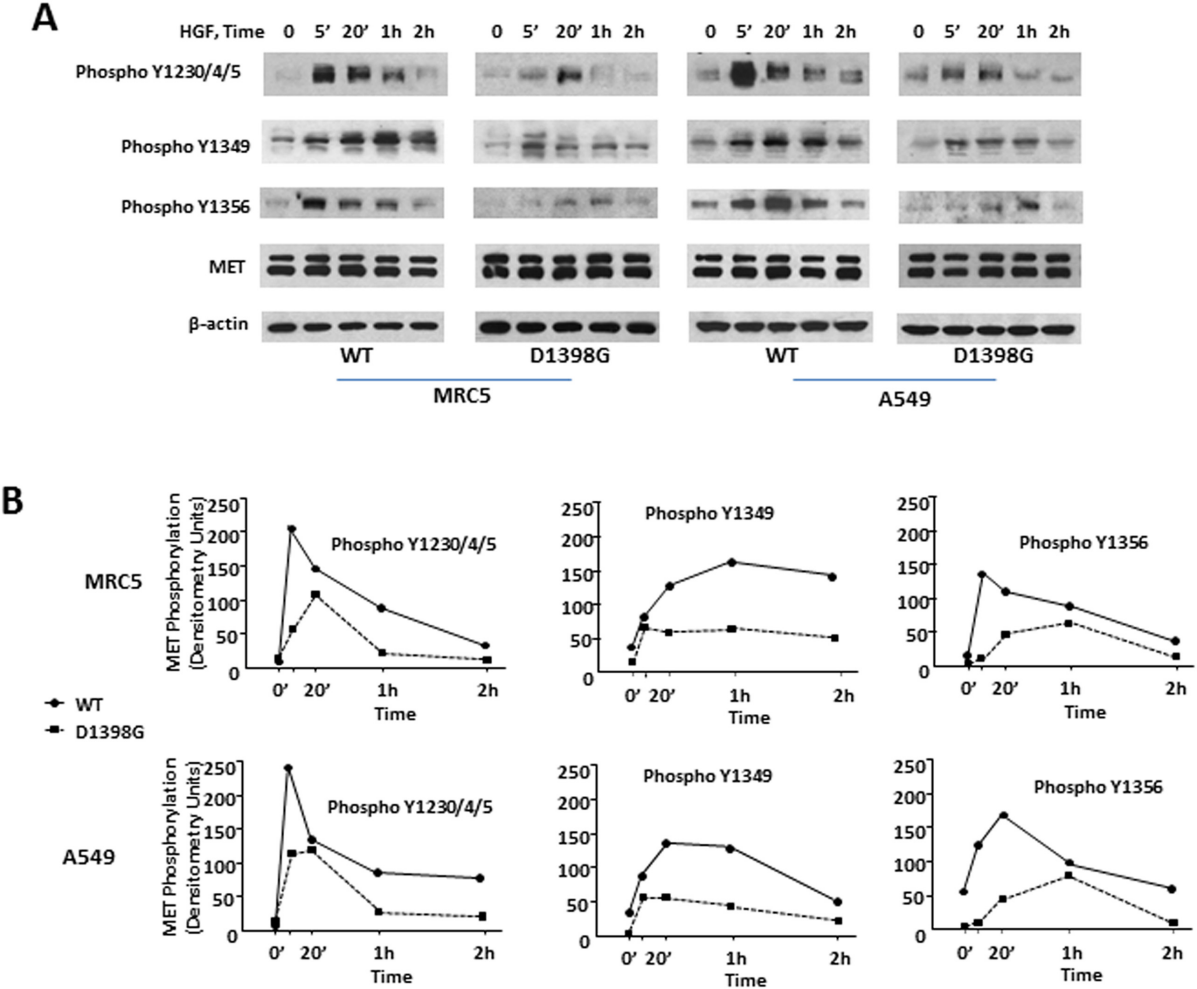
MET tyrosine phosphorylation in lung fibroblasts (LF) and A549 cells. **A.** Time course of MET WT and MET D1398G tyrosine phosphorylation was determined in LF and A549 incubated with HGF (50 ng/ml) for various time points. Cell extracts were immunoblotted with anti-phospho-MET or anti-MET polyclonal antibodies (see details in Materials and Methods). **B.** Quantitative results of densitometric analysis of immunoblots. Values are the mean and SD from three independent experiments.

As an initial approach to evaluate the functional consequences of D1398G mutation, we studied HGF-induced MET autophosphorylation in LF (MRC5 cell line) and AEC (A549 cell line) transfected either with MET WT or with D1398G mutant. MET tyrosine phosphorylation was assessed using anti-phospho-c-Met [pYpYpY^1230/1234/1235^], pY^1349^, and pY^1356^ antibodies. Treatment with HGF induced rapid phosphorylation of MET WT at tyrosine 1230, 1234, and 1235 in both LF and A549 cells. Maximal phosphorylation was observed within 5 minutes of HGF treatment and decreased by 20 minutes following HGF treatment (Fig 2). HGF-induced phosphorylation at tyrosine 1349 appeared to take place after tyrosine 1230, 1234, and 1235 phosphorylation with maximal levels observed between 20 minutes and 1 hour. The time course of HGF-induced phosphorylation at tyrosine 1356 in LF resembled tyrosine 1230, 1234, and 1235 phosphorylation with maximal levels observed within 5 minutes of HGF treatment. Tyrosine 1356 phosphorylation in A549 appeared to be stronger than in LF with maximal levels observed within 20 minutes of HGF treatment. In contrast to MET WT, tyrosine phosphorylation of MET D1398G was decreased and delayed in both LF and A549 cell lines. Thus, HGF-induced phosphorylation of MET D1398G at tyrosine 1356 was visibly reduced, not reaching its maximal value until 60 minutes as compared to 5 minutes for MET WT in LF and 20 minutes in A549 cells.

### Effects of HGF on collagen, SMA, and CCN2 in scleroderma and normal lung fibroblasts transfected with MET WT and MET D1398G

**Fig 3:**
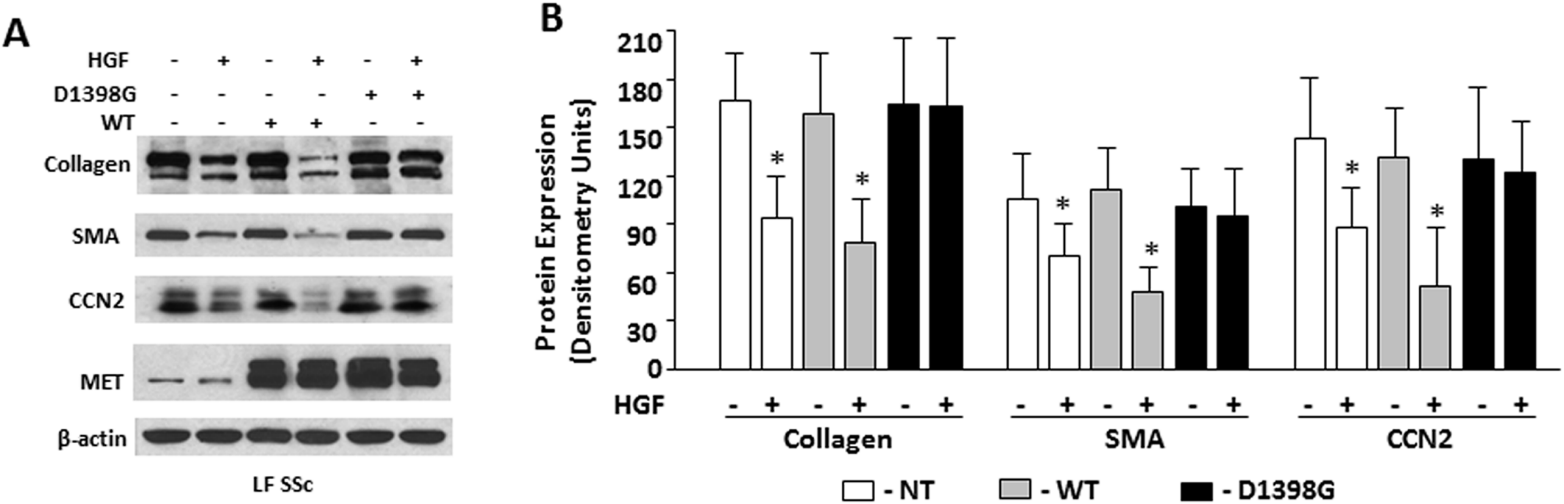
Expression of type I collagen, SMA, and CCN2 in scleroderma lung fibroblasts (LF) transfected with MET WT and MET D1398G. Non-transfected (NT) and transfected scleroderma (SSc) lung fibroblasts (LF) were serum-starved for 24 hours followed by incubation for 48 hours with recombinant HGF (50 ng/ml). The cells were collected with lysis buffer and analyzed by Western blot with indicated antibodies including MET as a transfection efficiency control. Anti-β-actin antibody was used as a loading control. **B.** Quantitative results of densitometric analysis of immunoblots. Values are the mean and SD from four independent experiments.

**Fig 4:**
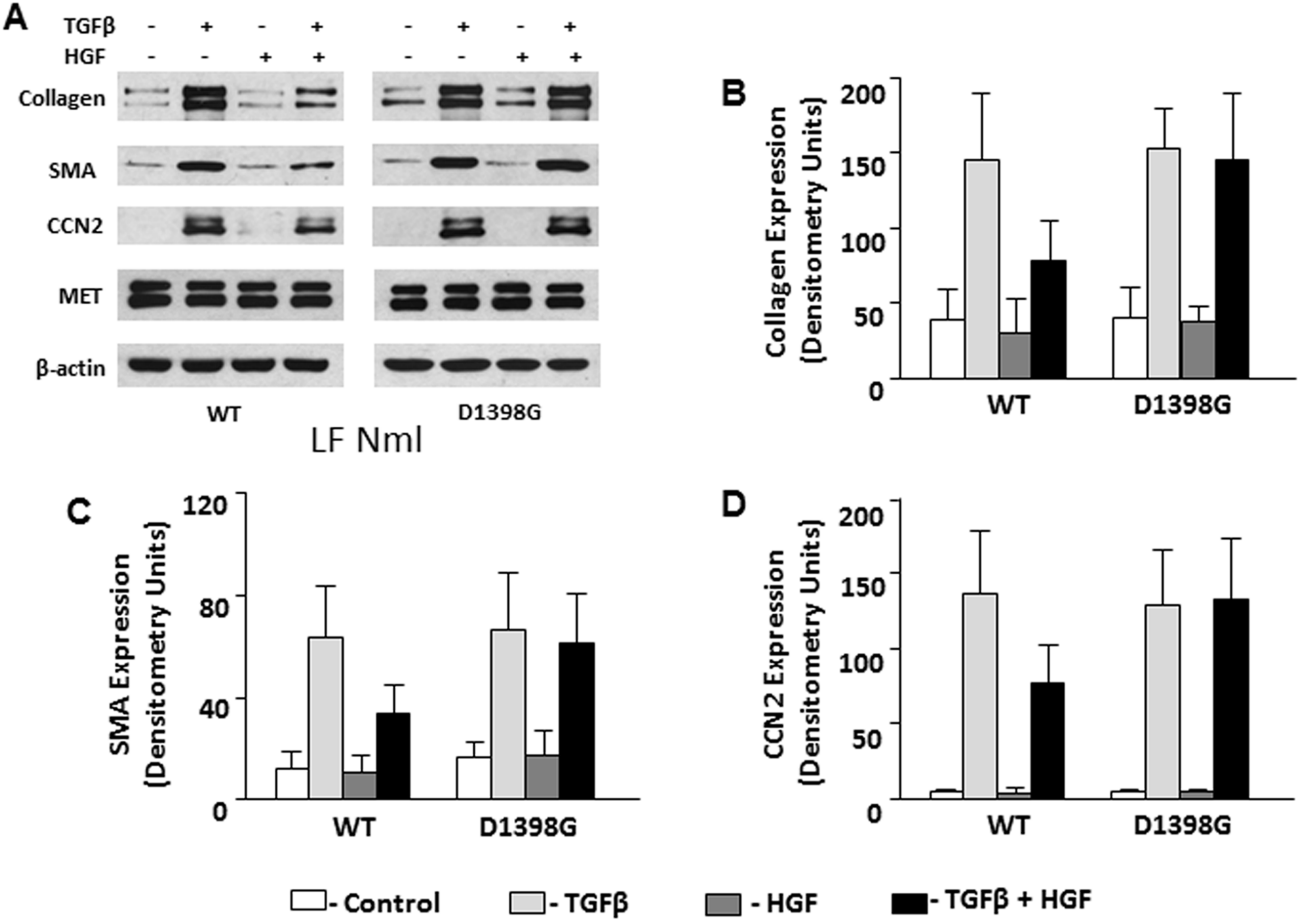
Expression of type I collagen, SMA, and CCN2 in normal lung fibroblasts (LF) transfected with MET WT and MET D1398G. Transfected lung fibroblasts were serum-starved for 24 h followed by incubation for 48 h with recombinant TGFβ (5 ng/ml) and/or HGF (50 ng/ml). The cells were collected with lysis buffer and analyzed by Western blot with indicated antibodies including MET as a transfection efficiency control and anti-β-actin antibody as a loading control. **B.** Densitometric analysis of immunoblots. Values are the mean and SD from three independent experiments.

Scleroderma LF transfected with the D1398G mutant or with the MET WT expressed similar to non-transfected cells basal levels of collagen, SMA, and CCN2. HGF, when added to cell culture medium at a concentration of 50 ng/ml for 48 hours, led to reduced expression of collagen, SMA, and CCN2 in non-transfected LF and LF transfected with MET WT, but not in LF transfected with mutant MET D1398G (Fig 3). To investigate the effects of D1398G mutation in normal LF, cells transfected with MET WT and mutant D1398G were pre-incubated with TGFβ followed by incubation with HGF for 48 hours. In normal LF, basal and TGFβ-induced collagen, SMA and CCN2 were indistinguishable between cells transfected with MET WT and mutant D1398G. HGF, however, decreased the levels of collagen, SMA and CCN2 in TGFβ-stimulated normal LF transfected with MET WT but not in TGFβ-stimulated normal LF transfected with MET D1398G (Fig 4).

### HGF-induced activation of Ras and MAPK in LF and A549 cells transfected with MET WT and MET D1398G

**Fig 5:**
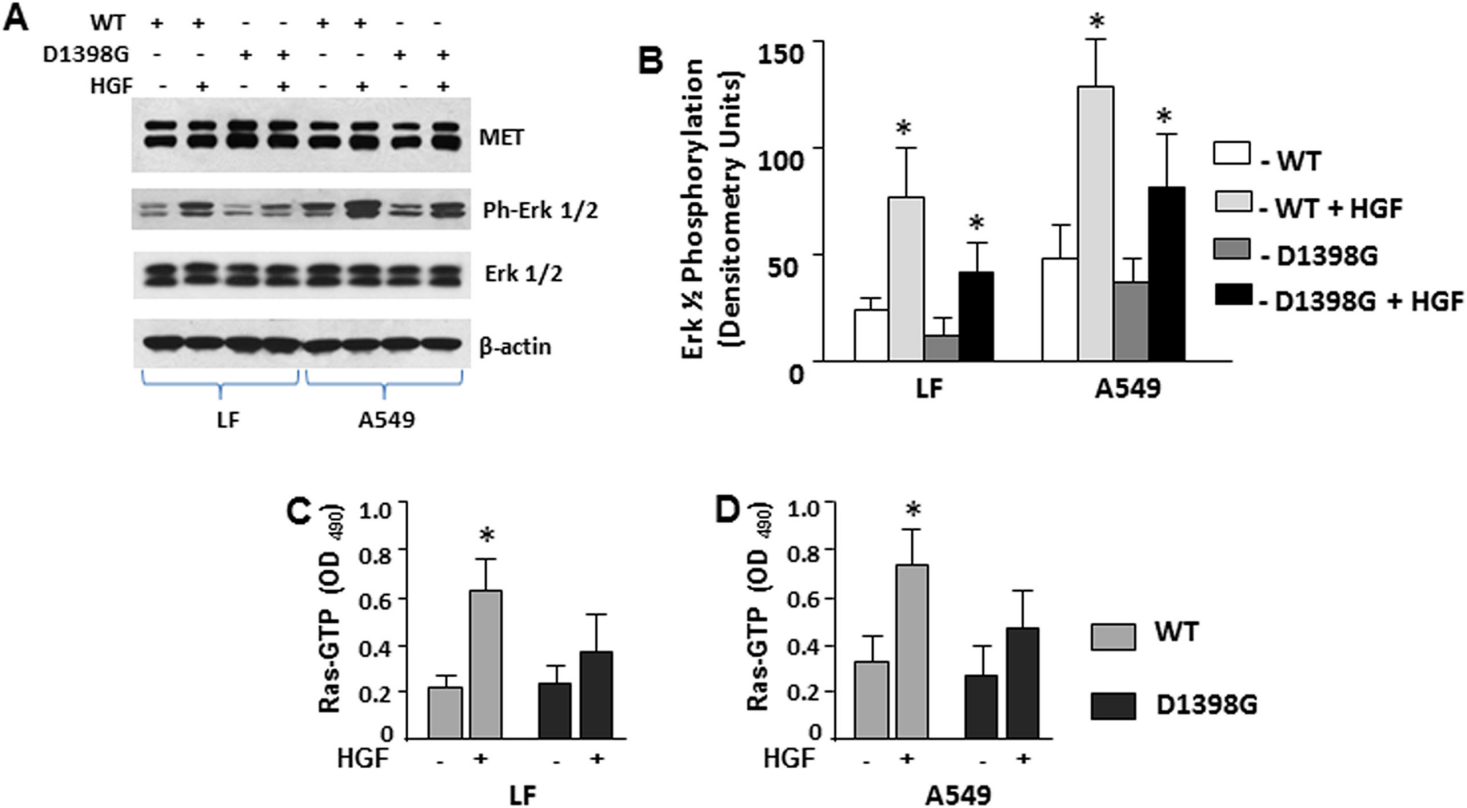
Effect of HGF on Ras activation and Erk1/2 phosphorylation in lung fibroblasts (LF) and A549 cells. **A.** HGF-induced Erk1/2 phosphorylation in LF and A549 cells transfected with MET WT and MET D1398G was determined in cells treated with HGF (50 ng/ml) for 10 min. Cell extracts were immunoblotted with anti-phospho-Erk1/2 or anti-Erk1/2, anti-MET, and anti-β-actin antibodies. **B.** Densitometric analysis of phospho-Erk1/2 immunoblots from three independent experiments. **C. and D.** Ras activity in LF (C) and A549 (D) transfected with MET WT and MET D1398G treated with and without HGF (50 ng/ml) for 10 min was determined as described under Materials and Methods. Values are the mean and SD from three independent experiments; *Statistically significant differences between cells stimulated with HGF versus control (p<0.05).

We previously demonstrated that HGF-induced down regulation of collagen and CCN2 in scleroderma LF is mediated by a MAPK-dependent pathway [25]. To investigate whether the D1398G mutation affects MAPK signaling pathways, we studied Erk1/2 phosphorylation. We noted that basal and HGF-induced Erk1/2 phosphorylation were more prominent in AEC as compared with LF. HGF further induced Erk1/2 phosphorylation in LF and AEC transfected either with MET WT or with MET D1398G. HGF-induced Erk1/2 phosphorylation in cells transfected with MET WT was notably higher as compared with cells transfected with MET D1398G (Fig 5A and 5B). The basal level of non-phosphorylated Erk1/2 in LF and AEC was not affected either by HGF stimulation or by transfection with D1398G mutation.

HGF-induced phosphorylation of Erk1/2 is mediated by Ras [30]. To investigate whether D1398G mutation affects MET-dependent Ras activation, we measured Ras-GTP in LF and in AEC transfected with MET WT and with MET D1398G. We observed a significant increase in total Ras activity in the presence of HGF in both LF and AEC transfected with MET WT, but not in either cell type transfected with MET D1398G (Fig 5C and 5D).

### D1398G prevents caspase-3 cleavage of the C-terminal portion of MET

**Fig 6:**
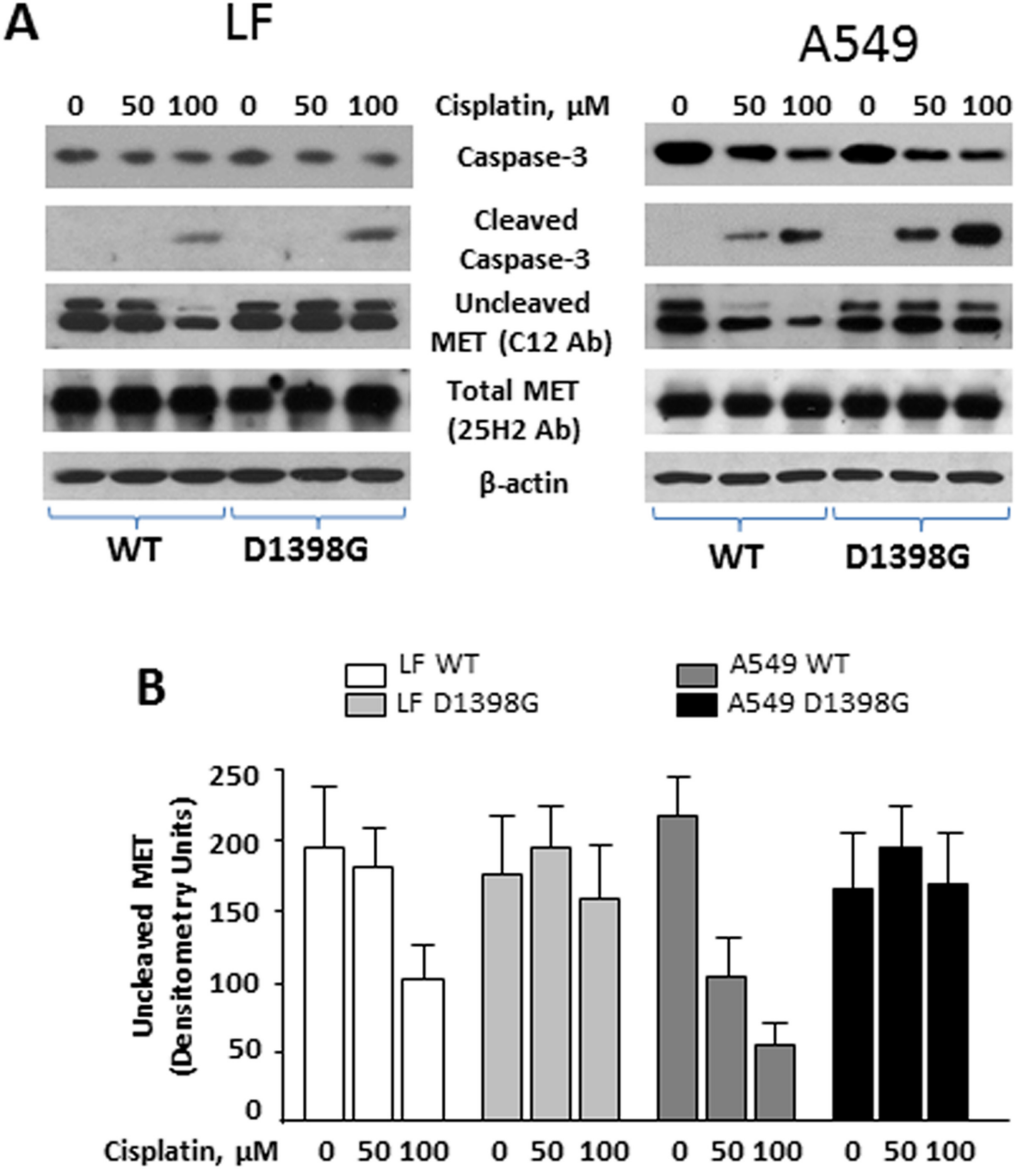
Effects of cisplatin in lung fibroblasts (LF) and A549 cells transfected with MET WT and MET D1398G. **A.** Transfected cells were incubated with or without cisplatin for 24 hours. The cells were collected with lysis buffer and analyzed by Western blot with indicated antibodies. **B.** Densitometric analysis of uncleaved MET detected by C12 antibody. Values are the mean and SD from three independent experiments.

Aspartic acid at position 1398 is the terminal amino acid of the caspase-3 cleavage motif, DEVD-T, at the C-terminus of MET (S2 Fig). To induce caspase-3 in cells, we used the caspase-3-activator cisplatin. We found AEC to be more sensitive to cisplatin treatment than LF. In fact, cleaved or active caspase-3 was detectable by Western blot in A549 and primary ATII but not in LF incubated with 50μM cisplatin for 24h. Further increases of cisplatin concentration (to 100μM) yielded cleaved caspase not only in A549 but also in LF (Fig 6). To investigate whether caspase-3 cleaves the C-terminal part of MET in A549 and LF, we employed various anti-MET antibodies that were generated against different parts of MET: C12 antibody was generated against the terminal 12 amino acids of MET, and 25H2 antibody was generated against the kinase domain of MET. Cisplatin-induced caspase-3 cleavage was associated with a loss of detectable MET protein in Western blots with the C12 antibody, suggesting that activated caspase-3 cleaves MET at position 1398. If TRPASFWETS, the terminal 10 amino acids of MET, are removed then C12 antibody no longer recognizes MET, resulting in decreased concentration of MET on Western blots. In contrast, under the same conditions MET protein levels detected by 25H2 antibody remain unchanged suggesting that C12 immunoblotting reflects loss of MET C-terminus but not the entire protein. Importantly, there was no difference in MET protein levels between C12 and 25H2 immunoblots of LF and A549 cells transfected with the D1398G mutant and incubated with and without cisplatin, thus indicating that caspase-3 is not able to recognize and cleave the DEVG-T motif.

### MET in lung tissues isolated from patients with SSc-ILD

**Fig 7:**
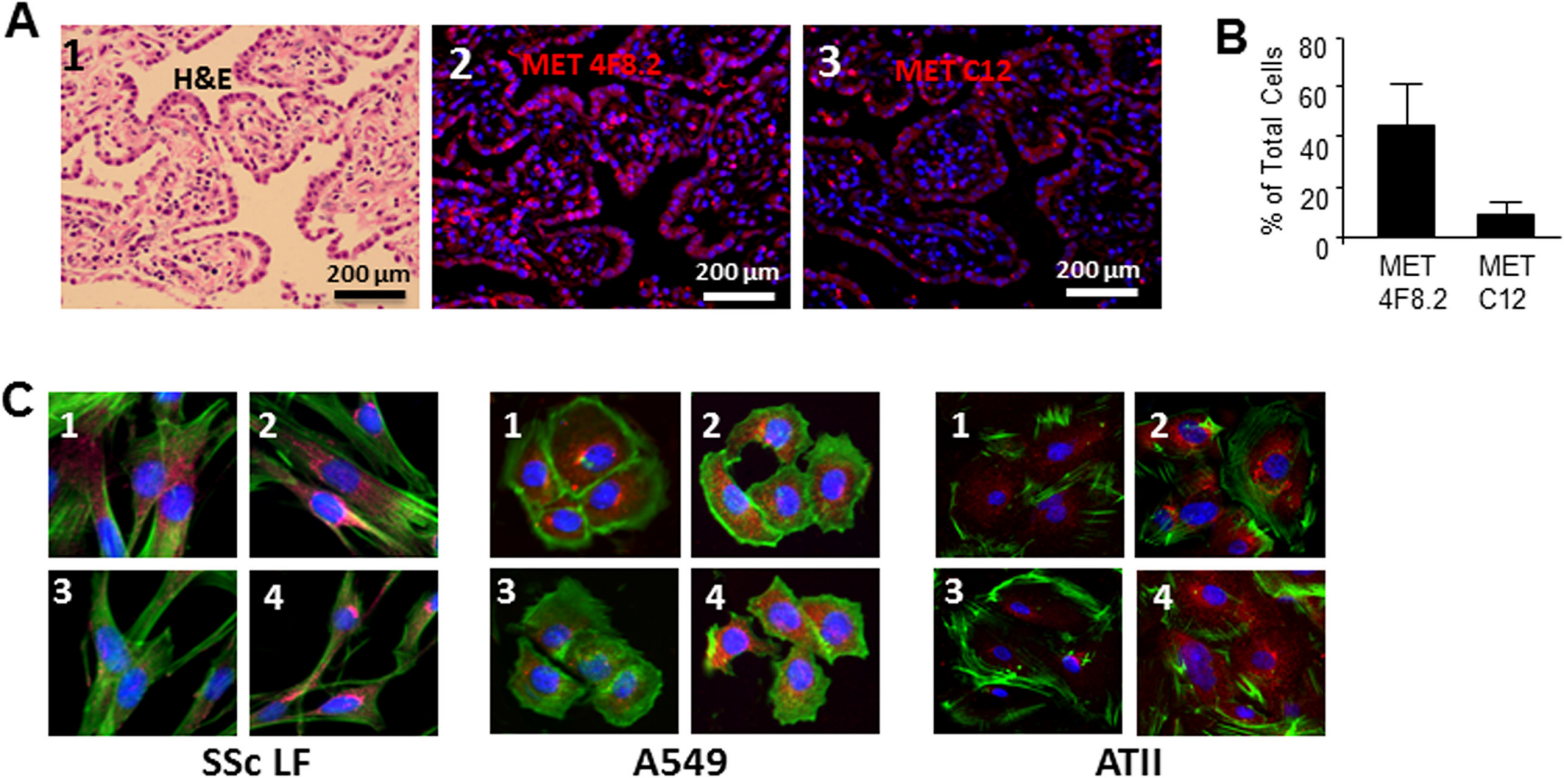
**A.** Expression of MET in lung tissues of patients with SSc-ILD. Panel 1 represents lung sections stained with hematoxylin and eosin (H&E). Panels 2 and 3 represent immunofluorescent images stained with anti-MET 4F8.2 antibody detecting total (cleaved and uncleaved MET) and anti-MET antibody C12 that does not recognize MET after C-terminal cleavage. Nuclei are stained with 4’,6-diamidino-2-phenylindole (DAPI). Representative images from three patients with SSc-ILD are presented. **B.** Quantitative results of image analysis for MET 4F8.2- and MET C12-positive cells. Cells (total, 4F8.2 positive and MET C12 positive) were counted on six randomly selected, no overlapping, high-power fields per sample at x400 magnification, and presented as mean and SD. **C.** Expression of endogenous MET in scleroderma lung fibroblasts (SSc LF), A549, and ATII cells. Cells were cultured in 4-chamber slides, challenged with (images 3 and 4) and without (images 1 and 2) cisplatin, fixed by 4% formaldehyde and stained with anti-MET 4F8.2 antibody (images 2 and 4) and anti-MET C12 antibody (images 1 and 3). Nuclei were stained with 4’,6-diamidino-2-phenylindole (DAPI). Phalloidin was used to show cytoskeleton.

To investigate whether loss of the MET C-terminus occurs in patients with SSc-ILD, we performed immunofluorescent staining of lung tissues with C12 antibody and with anti-MET 4F8.2 antibody generated against the MET extracellular domain. We studied lung tissues isolated from three different patients who died from end-stage SSc-ILD. Hematoxylin and eosin staining of each sample demonstrated severe disarrangement of lung architecture with thickened alveolar septae and residual air spaces (Fig 7A). In immunohistochemial studies, we observed that 44.5 ± 18.2% of cells in fibrotic lung tissues expressed MET as indicated by positive immunofluorescent signal with the 4F8.2 antibody. In contrast, only 9.1 ± 6.7% of total lung cells express the C-terminal portion of MET, suggesting that cleavage and loss of the MET C-terminus occurred in these SSc-ILD patients (Fig 7B).

To confirm that cleavage and loss of the MET C-terminus can occur with the endogenous MET, we used non-transfected scleroderma LF, A549, and ATII cell lines. To induce caspase-3 in cells, we employed the caspase-3-activator cisplatin in concentrations described above. Similarly to the described above immunofluorescent study of scleroderma lung tissue, we used anti-MET 4F8.2 antibody detecting total (cleaved and uncleaved MET) and anti-MET antibody C12 that does not recognize MET after C-terminal cleavage. After challenging cells with cisplatin, we observed a decrease of MET labeled with C12 antibody suggesting a loss of the terminal 10 amino acids of MET, TRPASFWETS. In contrast, amount of MET labeled with 4F8.2 antibody was not affected by cisplatin (Fig 7C).

### D1398G mutation interferes with anti-apoptotic effects of MET in AEC

**Fig 8:**
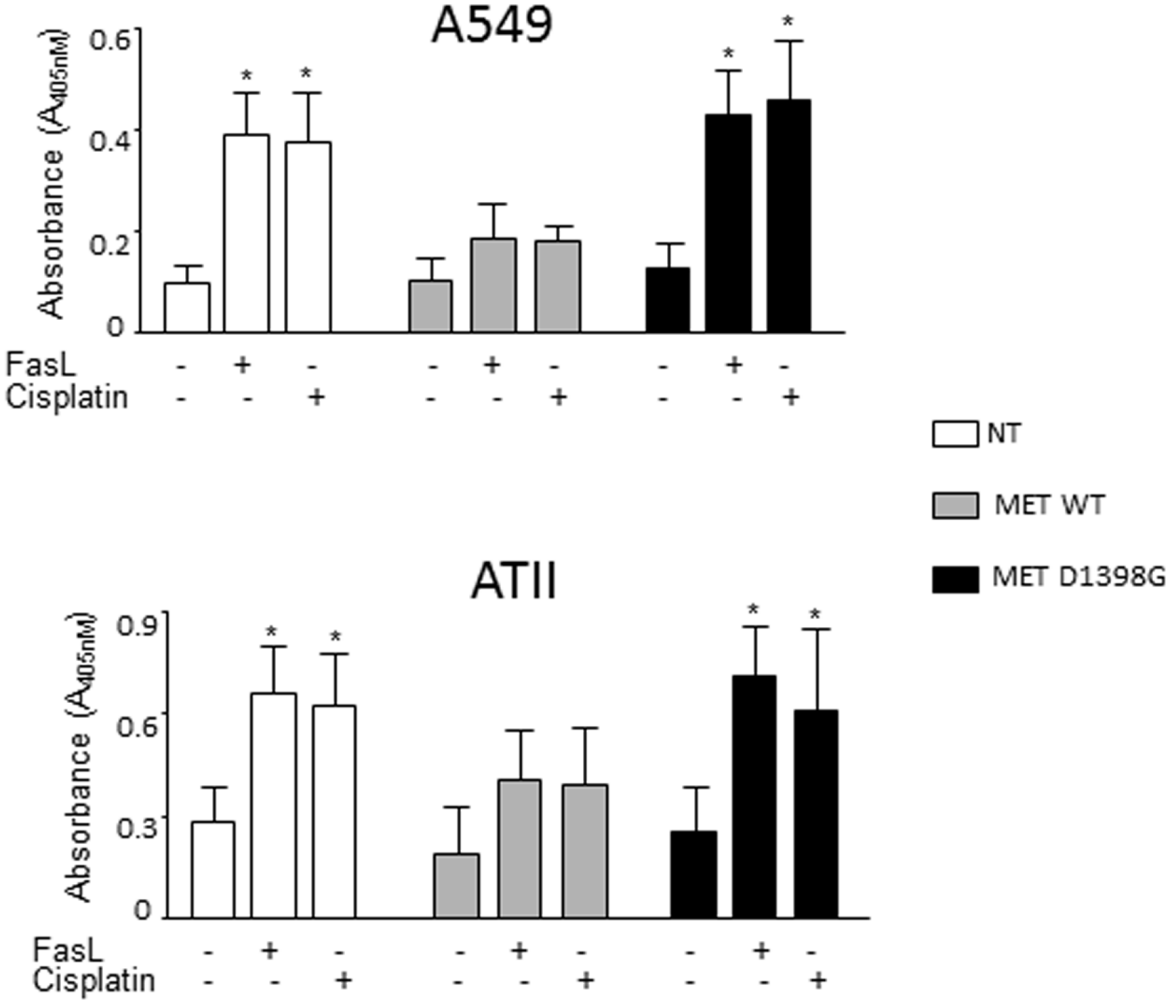
A549 and ATII cells transfected with MET WT but not with MET D1398G are protected from FasL- and cisplatin-induced apoptosis. Transfected cells were incubated with or without FasL or cisplatin for 24 hours, collected with lysis buffer and analyzed by Caspase 3 Assay as detailed in Materials and Methods. Each bar represents mean and SD of duplicate determinations in three experiments. NT—non-transfected. *Statistically significant differences FasL- and cisplatin-treated versus untreated cells, p<0.01.

A cell-protective effect of HGF and MET have been reported in AEC in numerous studies [31, 32]. To investigate if the D1398G mutation interferes with anti-apoptotic effects of MET in AEC; we induced apoptosis of A549 and primary AEC ATII with FasL and cisplatin and measured caspase-3 activity in cell lysates. Incubation of A549 and ATII cells for 24h with either FasL or cisplatin resulted in apoptosis reflected by an increase in the level of active caspase-3 (Fig 8). Transfection of A549 and ATII cells with MET WT significantly reduced apoptosis decreasing FasL- and cisplatin-induced caspase-3 activity (p < 0.01). In contrast, A549 and ATII cells transfected with the MET D1398G mutant were not protected from FasL- and cisplatin-induced apoptosis.

## Discussion

The MET gene is located on chromosome 7q21-q31 and encodes for a protein containing a 50 kDa extracellular α-chain and a transmembrane 140 kDa β-chain, which are linked by disulfide bonds [33]. In cancer, the MET gene readily acquires mutations within diseased tissues, and more than 20 missense or activating mutations have been described [34, 35]. However, none of the MET gene mutations has been identified in fibrotic diseases.

Following binding of HGF, under normal conditions MET undergoes auto-phosphorylation at tyrosine residues in its cytoplasmic domain and initiates a cascade of signal transduction events that lead to specific cellular responses [11, 36]. Aspartic acid (Asp, D) carries a hydrophilic group with strong negative charge located on the outer surface of protein. Asp (D) has been reported to be essential in several proteins for ligand recognition and phosphorylation [37, 38]. The major autophosphorylation sites in the tyrosine kinase domain of MET were identified as tyrosine residues 1230, 1234, and 1235 [13]. Here we demonstrate that MET phosphorylation on these residues, as well as on tyrosine residues 1349 and 1356 in the cytoplasmic domain, is delayed and decreased in both, MRC5 and A549, transfected with adenovirus carrying the D1398G mutant.

Accumulation of collagen, CCN2 and other extracellular matrix proteins in fibrotic lesions is a hallmark of scleroderma and other fibrotic diseases [39–41]. HGF has been shown to attenuate accumulation of collagen, CCN2 and SMA in several murine models of fibrosis [42, 43]. Previously, we demonstrated that the antifibrotic activity of HGF is different in LF isolated from African American and Caucasian scleroderma patients. In particular, HGF’s antifibrotic effect is reduced in LF isolated from African Americans and this is associated with decreased phosphorylation of MET [10]. The current observations are in line with our previous results, as cells transfected with the D1398G mutant demonstrated reduced MET phosphorylation in association with reduced antifibrotic activity of HGF.

In a previous study, we showed that HGF down-regulates collagen and CCN2 in lung fibroblasts through MAPK-dependent signaling pathways [25]. MAPK signaling is one of the major pathways induced by MET following HGF stimulation, tyrosine phosphorylation and Ras activation [25, 44, 45]. In the current study LF transfected with MET D1398G were characterized by decreased activation of Ras and reduced MAPK phosphorylation compared with LF transfected with MET WT, suggesting that these signaling pathways are affected by the D1398G mutation.

We recently described antifibrotic properties of the C-terminal fragment of MET M10 *in vitro* and *in vivo* [23]. Here we demonstrate that M10 is generated from the full-length MET by both AEC and LF. By utilizing an anti-MET antibody C12 that recognizes the terminal 12 amino acids of MET and does not detect MET when the M10 fragment is lacking, we show that the amount of MET detectable with C12 antibody on Western blot is decreased in a concentration-dependent manner after cisplatin treatment. On the other hand, the amount of MET detectable by 25H2 antibody, which detects MET with and without the M10 fragment, is not affected by cisplatin. Importantly, cells transfected with the MET D1398G mutant do not generate M10 since caspase-3 does not recognize glycine and cannot cleave MET C-terminus at 1398 position. As a result, the amount of MET detected by C12 antibody in cells transfected with D1398G is not affected by cisplatin.

Alveolar epithelium is the main source of MET in lung [46] and is the primary site of lung damage and activation of caspase-3 in SSc-ILD [47]. An increase in caspase-3 activity in SSc [47] suggests that *in vivo* cleavage of MET can occur. Indeed, immunofluorescent studies using antibodies that differentially recognize MET expressing or not expressing the terminal 10 amino acids (TRPASFWETS) support the notion that such cleavage can indeed occur *in vivo*.

In addition to downregulating collagen and other extracellular matrix proteins, antifibrotic properties of HGF and MET are based also on anti-apoptotic effects in AEC [42, 43, 46]. We found that overexpression of MET WT, but not variant MET D1398G, protects AEC from cisplatin- and FasL-induced apoptosis, which suggests that in order to initiate cell-protective effects, MET must be cleaved at the 1398 aspartic acid. Anti-apoptotic mechanisms of HGF-activated MET, based on PI3K-Akt and Ras-Erk signaling pathways, have been previously elucidated [32, 46]. Here we report that MET WT, but not the D1398G mutant, when overexpressed, protects A549 and ATII cells from apoptosis even without HGF, suggesting that either the TRPASFWETS fragment or the new C-terminus, DEVD, might play a role in an HGF-independent anti-apoptotic mechanism of MET in AEC.

Our study demonstrates that D1398G mutation in MET is associated with compromised HGF signaling in lung fibroblasts and in AEC because of decreased auto-phosphorylation and reduced activation of Ras and MAPK signaling. Generation of the C-terminal fragment of MET requires aspartic acid at position 1398 and can be in part responsible for anti-apoptotic and antifibrotic effects of MET.

## Supporting Information

S1 Fig Generation of recombinant MET wild type and MET D1398G adenoviruses. (DOCX)

S2 Fig Real-time RT-PCR analysis of pro-SPC expression in A549 cells. (DOCX)

S3 Fig Ethidium bromide stained agarose gel demonstrating the purity of GAPDH- and pro-SPC-specific transcripts after real-time PCR (40 cycles). (DOCX)

S4 Fig Location of aspartic acid at the position of 1398 and peptide TRPASFWETS at the MET receptor tyrosine kinase. (DOCX)
