# Supplementary figures and images for: D1398G Variant of MET Is Associated with Impaired Signaling of Hepatocyte Growth Factor in Alveolar Epithelial Cells and Lung Fibroblasts
Source: PLoS One. 2016 Sep 1;11(9):e0162357. doi: 10.1371/journal.pone.0162357 (PMC5008815; doi:10.1371/journal.pone.0162357)

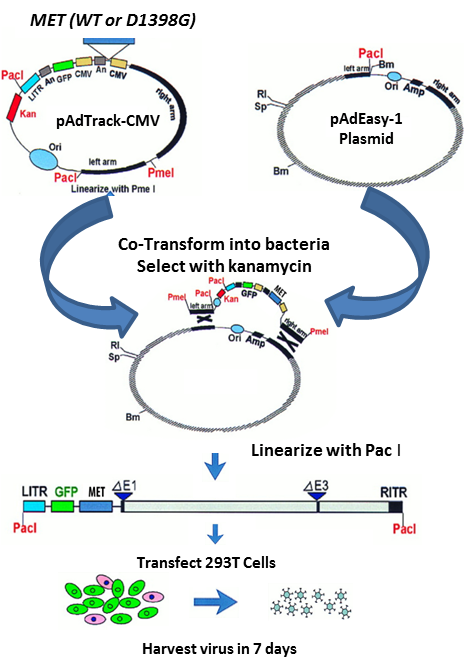


Figure S1. **Generation of recombinant MET wild type and MET D1398G adenoviruses.**

Supplement: S1 Fig — (DOCX) [file pone.0162357.s001.docx]
