# Supplementary material for: D1398G Variant of MET Is Associated with Impaired Signaling of Hepatocyte Growth Factor in Alveolar Epithelial Cells and Lung Fibroblasts
Source: PLoS One. 2016 Sep 1;11(9):e0162357. doi: 10.1371/journal.pone.0162357 (PMC5008815; doi:10.1371/journal.pone.0162357)
Supplement: S2 Fig — (DOCX) [file pone.0162357.s002.docx]

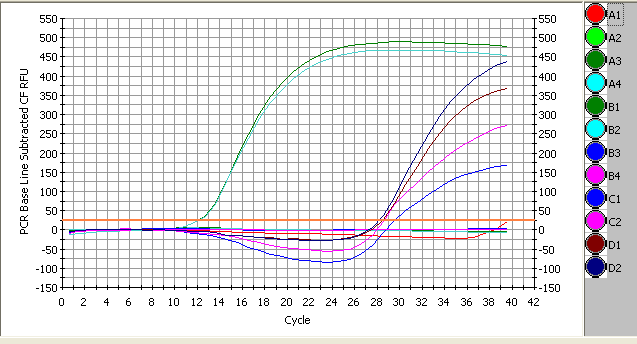


GAPDH

Pro-SPC

(SFM)

Pro-SPC

(10% FBS)

Negative controls

Threshold line

Figure S2. **Real-time** **RT-PCR analysis of pro-SPC expression in A549 cells.** Real-time amplification plots of pro-SPC expression in A549 cells cultured in serum-free medium (SFM) and cells cultured in medium supplemented with serum (10% FBS) are presented. Curve-fit relative fluorescence units (CF RFU) serves as a function of PCR cycle number. The horizontal line at RFU = 0.25 is the threshold for detection. GAPDH is used as a housekeeping gene.
