# Supplementary material for: D1398G Variant of MET Is Associated with Impaired Signaling of Hepatocyte Growth Factor in Alveolar Epithelial Cells and Lung Fibroblasts
Source: PLoS One. 2016 Sep 1;11(9):e0162357. doi: 10.1371/journal.pone.0162357 (PMC5008815; doi:10.1371/journal.pone.0162357)
Supplement: S3 Fig — (DOCX) [file pone.0162357.s003.docx]

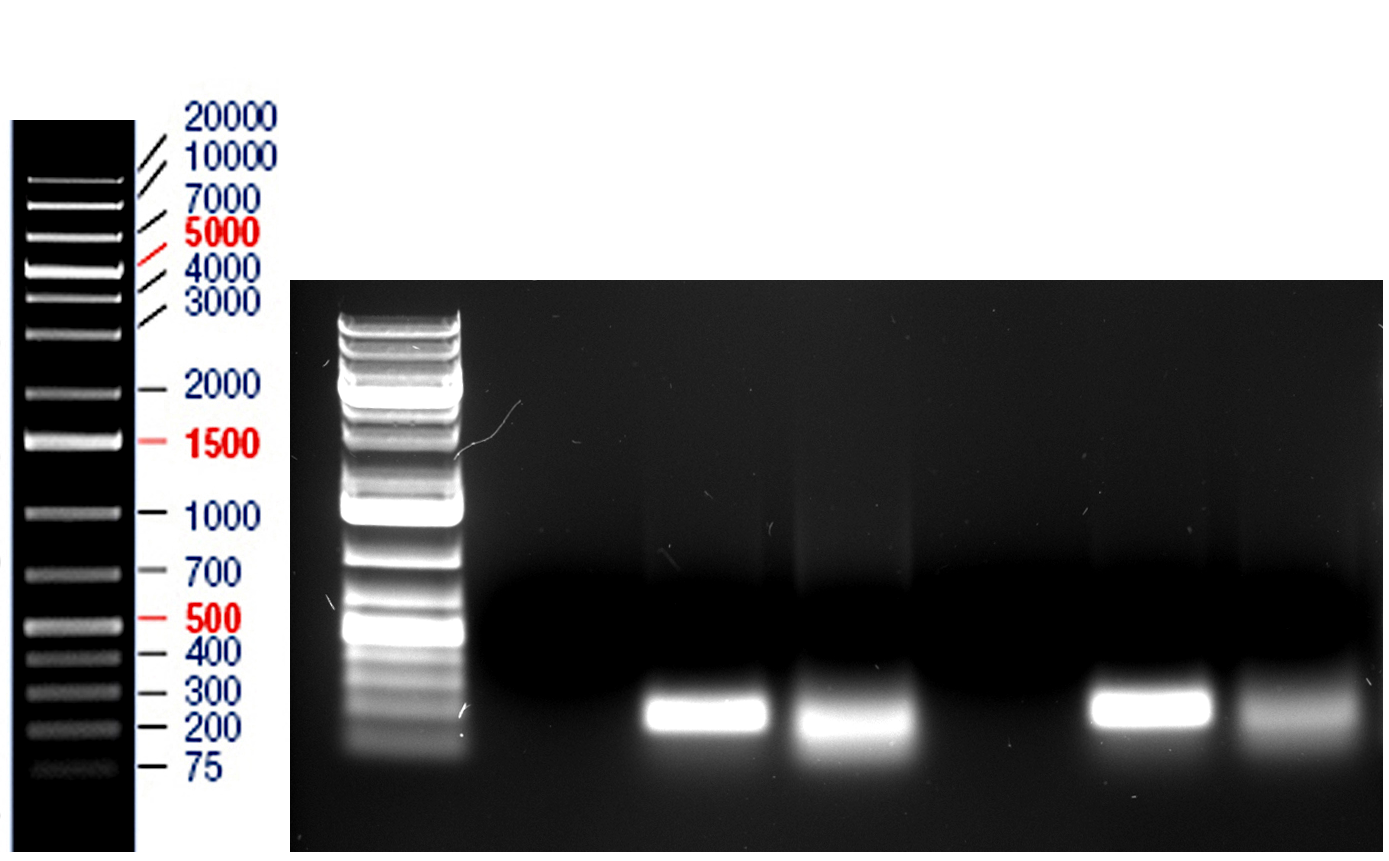


1 2 3 4 5 6

SFM 10% FBS

Figure S3. **Ethidium bromide stained agarose gel demonstrating the purity of GAPDH- and pro-SPC-specific transcripts after real-time PCR (40 cycles).** Lanes 1 and 4 represent pro-SPC negative control; lanes 2 and 5 represent GAPDH-specific transcript (167 bp); lanes 3 and 6 represent pro-SPC-specific transcript (134 bp).
