# Supplementary material for: D1398G Variant of MET Is Associated with Impaired Signaling of Hepatocyte Growth Factor in Alveolar Epithelial Cells and Lung Fibroblasts
Source: PLoS One. 2016 Sep 1;11(9):e0162357. doi: 10.1371/journal.pone.0162357 (PMC5008815; doi:10.1371/journal.pone.0162357)
Supplement: S4 Fig — (DOCX) [file pone.0162357.s004.docx]

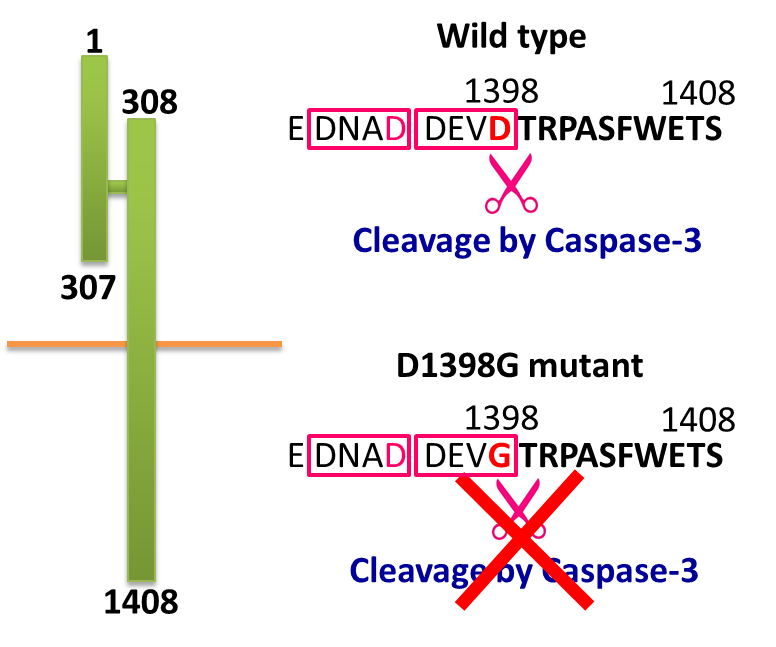


Figure S4. **Location of aspartic acid at the position of 1398 and peptide TRPASFWETS at the MET receptor tyrosine kinase.**
